# Horizontally Acquired nrDNAs Persist in Low Amounts in Host *Hordeum* Genomes and Evolve Independently of Native nrDNA

**DOI:** 10.3389/fpls.2021.672879

**Published:** 2021-05-17

**Authors:** Karol Krak, Petra Caklová, David Kopecký, Frank R. Blattner, Václav Mahelka

**Affiliations:** ^1^Czech Academy of Sciences, Institute of Botany, Prùhonice, Czechia; ^2^Faculty of Environmental Sciences, Czech University of Life Sciences Prague, Prague 6, Czechia; ^3^Czech Academy of Sciences, Institute of Experimental Botany, Centre of the Region Haná for Biotechnological and Agricultural Research, Olomouc, Czechia; ^4^Experimental Taxonomy, Leibniz Institute of Plant Genetics and Crop Plant Research, Gatersleben, Germany; ^5^German Centre of Integrative Biodiversity Research (iDiv) Halle–Jena–Leipzig, Leipzig, Germany

**Keywords:** copy number variation (CNV), horizontal gene transfer (HGT), internal transcribed spacer (ITS), nuclear ribosomal DNA (nrDNA), fluorescent *in situ* hybridisation (FISH), phylogeny, qPCR (quantitative PCR), xenolog

## Abstract

Nuclear ribosomal DNA (nrDNA) has displayed extraordinary dynamics during the evolution of plant species. However, the patterns and evolutionary significance of nrDNA array expansion or contraction are still relatively unknown. Moreover, only little is known of the fate of minority nrDNA copies acquired between species via horizontal transfer. The barley genus *Hordeum* (Poaceae) represents a good model for such a study, as species of section *Stenostachys* acquired nrDNA via horizontal transfer from at least five different panicoid genera, causing long-term co-existence of native (*Hordeum*-like) and non-native (panicoid) nrDNAs. Using quantitative PCR, we investigated copy number variation (CNV) of nrDNA in the diploid representatives of the genus *Hordeum*. We estimated the copy number of the foreign, as well as of the native ITS types (ribotypes), and followed the pattern of their CNV in relation to the genus’ phylogeny, species’ genomes size and the number of nrDNA loci. For the native ribotype, we encountered an almost 19-fold variation in the mean copy number among the taxa analysed, ranging from 1689 copies (per 2C content) in *H. patagonicum* subsp. *mustersii* to 31342 copies in *H. murinum* subsp. *glaucum*. The copy numbers did not correlate with any of the genus’ phylogeny, the species’ genome size or the number of nrDNA loci. The CNV was high within the recognised groups (up to 13.2 × in the American I-genome species) as well as between accessions of the same species (up to 4×). Foreign ribotypes represent only a small fraction of the total number of nrDNA copies. Their copy numbers ranged from single units to tens and rarely hundreds of copies. They amounted, on average, to between 0.1% (*Setaria* ribotype) and 1.9% (*Euclasta* ribotype) of total nrDNA. None of the foreign ribotypes showed significant differences with respect to phylogenetic groups recognised within the sect. *Stenostachys*. Overall, no correlation was found between copy numbers of native and foreign nrDNAs suggesting the sequestration and independent evolution of native and non-native nrDNA arrays. Therefore, foreign nrDNA in *Hordeum* likely poses a dead-end by-product of horizontal gene transfer events.

## Introduction

Ribosomal RNA (rRNA) is an essential structural component of ribosomes, the sites of protein synthesis. Ribosomes consist of two subunits, each of which is composed of several proteins and rRNA molecules. In eukaryotes, the large subunit consists of three rRNA molecules (25–26S, 5.8S and 5S), whereas the small subunit includes just one rRNA (18S) molecule (Ben-Shem et al., 2011). High demand for ribosomal RNA needed for ribosome assembly is satisfied through the transcription of numerous copies of the nuclear ribosomal (nrDNA) genes. Three of the four rRNA genes in eukaryotes, coding for 18S, 5.8S, and 26S (altogether referred to as 35S), are separated by two internal transcribed spacers (ITS) and together constitute a single transcription unit (Potapova and Gerton, 2019). Transcription units are separated from one another by intergenic spacers (IGS). Plant genomes harbour thousands of nrDNA transcription units, which are organised in large tandem arrays forming the so-called nucleolar organiser regions (NORs) located on a variable number of chromosomes (Srivastava and Schlessinger, 1991; Dubcovsky and Dvořák, 1995).

The number of nrDNA copies has been found to correlate with genome size at large scale in both plants and animals (Prokopowich et al., 2003). Nevertheless, at lower taxonomic levels this relationship is largely unexplored. The extent of the nrDNA copy number variation (CNV) depends on the group studied. It is somewhat conserved among liverworts, mosses and hornworts (Rosato et al., 2016). Conservation is highly variable among species (Cronn et al., 1996) as well as within species of seed plants (Govindaraju and Cullis, 1992; Malinská et al., 2010), fungi (Johnson et al., 2015) and vertebrates (Veiko et al., 2007). Despite the fact that nrDNA occupies a significant portion of the eukaryotic genome, the relationship between species-level phylogeny and nrDNA copy number has barely been investigated (Sproul et al., 2020).

Nuclear ribosomal DNA is an exemplar member of a multigene family and known for its ability to maintain sequence homogeneity. The nrDNA units change their sequences in a highly synchronised manner – described as concerted evolution (Arnheim et al., 1980). Notwithstanding, nrDNAs of different origin have been found to coexist within a single genome as a result of hybridisation and allopolyploidisation events (Álvarez and Wendel, 2003). The patterns of sequence evolution and array contraction and/or expansion of the putative parental copies, co-existing within the hybrid genomes, are well explored (Matyášek et al., 2007; Malinská et al., 2010). The fates of the parental ribotypes range from complete homogenisation (Wendel et al., 1995; Fuertes Aguilar et al., 1999) over independent evolution of parental sequences and their maintenance at different abundances (e.g., Malinská et al., 2010) to the occurrence of newly arising recombinant ribotypes (e.g., Ko and Jung, 2002). Horizontal gene transfer (HGT) represents another mechanism that can contribute to increased diversity of nrDNA within a single genome. However, the evolutionary dynamics of nrDNA sequences acquired via HGT has not been investigated.

Recently, Mahelka et al. (2017) described extensive HGT involving nrDNA. They found that wild barley (*Hordeum*, Pooideae) species possess, in addition to their native nrDNA copies, nrDNA sequences that correspond to grasses from the subfamily Panicoideae. The transferred nrDNAs occur only in the I-genome *Hordeum* taxa (= sect. *Stenostachys*; for infrageneric classification of *Hordeum* see Blattner, 2009; Brassac and Blattner, 2015), and certain wild barley species (and individuals) possess non-native nrDNAs from up to five panicoid donors (namely *Arundinela*, *Euclasta*, *Panicum*, *Paspalum*, and *Setaria*). Phylogenetic patterns suggest the acquisition of the panicoid DNA occurred via at least nine independent horizontal transfers within a timeframe of between 5 and 1 mya. Based on substitution patterns within the ITS region and the absence of mRNA expression, the authors considered the foreign ribotypes as pseudogenes (Mahelka et al., 2017). In a follow-up study, Mahelka et al. (2021) focused specifically on the transfer from a *Panicum*-like donor into *Hordeum* that is likely the oldest of the nine transfers between panicoid grasses and *Hordeum*. It predated the diversification of sect. *Stenostachys*, and occurred between 5 and 1.7 mya. Along with several protein-coding genes and transposable elements, these authors show that the *Panicum*-like ribotypes resided within a specific *Panicum*-derived chromosomal segment, which is located on a NOR-bearing chromosome, although on the opposite chromosome arm in various *Hordeum* species from sect. *Stenostachys*.

Mahelka et al. (2017) carried out a detailed characterisation of the non-native genetic material at the level of sequence variation in a phylogenetic context, but did not focus on quantification of the foreign nrDNAs in the *Hordeum* genomes. The number of particular foreign ribotypes in the *Hordeum* genomes – in terms of copy number, coupled with within – as well as between-species dynamics – remains an unanswered questions.

The objective of our study is to investigate the CNV of nrDNA in diploid representatives of the genus *Hordeum*. In the main, we ask what is the CNV of the foreign ribotypes in the I-genome species? Also, whether, and how, the CNV correlates with the CNV of native ribotypes in *Hordeum*? To answer these questions, we investigated the following: (i) the CNV of the native ribotype in diploid species representing the entire genus *Hordeum*; (ii) the CNV of particular foreign ribotypes in species of section *Stenostachys*; (iii) any correlations in the patterns of CNV between the native and foreign ribotypes; (iv) any correlation between the CNV of both native and foreign ribotypes and species-level phylogeny, genome size and the number of nrDNA loci. We hypothesise that a minor proportion of foreign ribotypes in the total nrDNA, coupled with independent evolution of foreign and native ribotypes, suggest the persistence of foreign nrDNAs within minor nrDNA loci aside from the main and active nrDNA loci (NORs). Together with other lines of evidence, such as non-functionality of the foreign nrDNAs and their confirmed occurrences in chromosomal parts located outside the NORs (Mahelka et al., 2017, 2021), the above scenario would constitute additional support for the status of foreign nrDNAs in *Hordeum* as dead-end by-products of HGT events.

## Materials and Methods

### Plant Material and DNA Quality Check

The plant material and high-quality DNA used in this study was available from a previous study (Mahelka et al., 2017). Details of the plant material is provided in Table 1 and Supplementary Table 1, for further details on the origins of particular accessions see Mahelka et al. (2017). The DNA of all samples was checked for integrity on agarose gel, and quantified using Qubit fluorometer (Thermo Fisher) with the Quibit dsDNA HS Assay kit (Thermo Fisher) according to the manufacturer’s instructions.

**TABLE 1:** Copy numbers of native and foreign ribotypes in *Hordeum* as estimated using qPCR.

Taxon	Geography	Clade (Subclade)	Genome size (pg/2C)	No. of loci (pairs)	Mean ribotype copy number (s.d.) per 2C				
					Native	*Panicum*	*Paspalum*	*Setaria*	*Euclasta*
*H. bogdanii*	Central Asia	I (Eurasian)	9.48	1*	9320 (1989)	17 (5)	n.d.	n.d.	n.d.
*H. californicum*	North America	I (American, californicum)	8.19	1*	8478 (2516)	12 (8)	n.d.	n.d.	19 (8)
*H. chilense*	Central Argentina	I (American, “core” species)	8.77	2	21665	36	98	n.d.	n.d.
*H. comosum*	Patagonia	I (American, “core” species)	8.97	n.d.	3929	10	12	4	60
*H. cordobense*	Central Argentina	I (American, muticum/cordobense)	9.19	2*	4122	14	9	n.d.	14
*H. erectifolium*	Central Argentina	I (American, “core” species)	9.49	2*	22282	n.d.	45	4	n.d.
*H. euclaston*	Central Argentina	I (American, “core” species)	6.85	2*	6416 (1798)	n.d.	134 (177)	n.d.	n.d.
*H. flexuosum*	Central Argentina	I (American, “core” species)	8.51	2*	13980	16	59	n.d.	n.d.
*H. intercedens*	North America	I (American, “core” species)	7.01	2*	12779 (10846)	n.d.	9 (9)	n.d.	n.d.
*H. muticum*	Central Argentina	I (American, muticum/cordobense)	9.57	1*	3498 (1933)	15 (7)	n.d.	n.d.	28 (31)
*H. patagonicum* subsp. *magellanicum*	Patagonia	I (American, “core” species)	9.33	2	13212	25	39	n.d.	73
*H. patagonicum* subsp. *mustersii*	Patagonia	I (American, “core” species)	8.77	2	1689	15	18	n.d.	n.d.
*H. patagonicum* subsp. *patagonicum*	Patagonia	I (American, “core” species)	9.46	2	2337 (291)	36 (26)	59 (27)	n.d.	132
*H. patagonicum* subsp. *setifolium*	Patagonia	I (American, “core” species)	9.61	2*	10996 (6986)	23 (3)	10 (8)	13	134 (20)
*H. pubiflorum*	Patagonia	I (American, “core” species)	8.70	2*	3830 (2727)	23 (8)	6 (3)	n.d.	26 (27)
*H. pusillum*	North America	I (American, “core” species)	7.16	2*	12488 (2802)	n.d.	401	n.d.	n.d.
*H. roshevitzii*	Central Asia	I (Eurasian)	9.69	1*	11138 (880)	18 (9)	n.d.	n.d.	n.d.
*H. stenostachys*	Central Argentina	I (American, “core” species)	9.38	2*	7257 (4683)	26	50 (38)	16 (7)	474
*H. gussoneanum*	Western Eurasia	Xa	10.41	1	5031 (3101)	n.d.	n.d.	n.d.	n.d.
*H. marinum*	Western Eurasia	Xa	9.10	1*	14026 (8769)	n.d.	n.d.	n.d.	n.d.
*H. murinum* subsp. *glaucum*	Western Eurasia	Xu	9.11	2*	31342 (13353)	n.d.	n.d.	n.d.	n.d.
*H. vulgare* subsp. *spontaneum*	Western Eurasia	H	10.59	2	24415	n.d.	n.d.	n.d.	n.d.
*H. vulgare subsp. vulgare*	Western Eurasia	H	10.59	2	26556	n.d.	n.d.	n.d.	n.d.

Standard deviation (brackets) is given for taxa with two accession analysed. Only diploid taxa were analysed. Geography – geographic distribution; Clade – phylogenetic position within Hordeum; the I-genome species are further divided into monophyletic subclades based on Brassac and Blattner (2015); Genome size values are from Jakob et al. (2004); * numbers of loci identified in this study (for details see Supplementary Table 1 and Supplementary Figures 3, 4); n.d. = ribotype not detected from the sample.

### Preparation of Standards for qPCR Estimation

Estimation of copy number using qPCR, requires the parallel measurement of standards to control qPCR efficiency. We employed results from our previous study (Mahelka et al., 2017), in which bacterial colonies containing all of the different ribotypes (both native and foreign) were stored as deep-frozen glycerol stocks. From this source we retrieved the relevant representative samples, and from these we isolated the plasmids using the Qiagen Plasmid Mini Kit (Qiagen) according to manufacturer’s instructions. The plasmids were linearised using an appropriate restriction enzyme with a single recognition site in the plasmid region. The linearised plasmids were purified using the Qiaquick PCR Purification Kit (Qiagen) and their concentrations were measured with a Qubit fluorometer using the Quibit dsDNA HS Assay kit (Thermo Fisher). The plasmids were then diluted to a concentration of 1 ng/μl. Copy numbers of the target ribotypes were calculated for the amplified fragments using the following equation: Copy number (ng^–1^) DNA = (6.022 × 10^23^)/(L × 10^–9^ × 660), where L is the length in bp of the amplified fragment (e.g., the insert), as implemented in the online tool^1^. Lengths of the inserts were as follows: native (*Hordeum*-like) 644 bp, *Panicum*-like ribotype 538 bp, *Paspalum*-like ribotype 537 bp, *Setaria*-like ribotype 539 bp, and *Euclasta*-like ribotype 536 bp. The plasmids were serially diluted to obtain a range of copies of the target ribotypes between 10^6^ and 1 (in a 10-fold dilution series) and these dilution series were used for the qPCR efficiency estimates.

### Development and Testing of qPCR Assays

The qPCR assays, targeting the ITS1-5.8S-ITS2 region of nrDNA, were developed based on the sequence variation available from Mahelka et al. (2017). We targeted the primers to the regions showing the highest specificity for each ribotype. At first, the specificity of the primers was tested *in silico* using Geneious 10.2.6 (Biomatters Ltd.), later, specificity was tested using the ribotype-specific plasmid standards. Each standard was amplified by all qPCR assays. Assays showing cross-amplification were further redesigned and tested, until only specific amplification of the target ribotype was obtained. Further, the reaction conditions of each assay were optimised using the serial dilutions of the respective plasmid standards in order to obtain maximal amplification efficiency.

### Limitation of the qPCR Assays

Our initial intention was to develop seven qPCR assays: five targeting the foreign ribotypes as found in *Hordeum*, e.g., *Panicum*-, *Paspalum*-, *Euclasta*-, *Setaria*-, and *Arundinella*-like (Mahelka et al., 2017), one assay specific for the native *Hordeum* ribotype and a universal assay that would amplify the native as well as the foreign ribotypes (total nrDNA). Unfortunately, the overall pattern of sequence variation hindered the development of an assay specific for *Arundinella* and the assay targeting the native *Hordeum* ribotype. Hence, we use the universal assay not only to estimate the copy number of total nrDNAs but also to estimate the copy number of the native ribotype. Therefore, in species of sect. *Stenostachys*, an overestimate of native ribotype is likely. However, given the low proportion of foreign ribotypes among the total nrDNAs, the bias is considered unlikely to be serious. In the species outside this section (*H. vulgare, H. marinum, H. murinum, H. gussoneanum*) that lack the foreign ribotypes, the estimate is likely to be unbiased.

### Estimation of nrDNA Copy Number Using qPCR

All qPCRs were carried out using the LightCycler 480 II Real-Time PCR instrument (Roche). To quantify the native ribotype, a TaqMan probe-based assay with the Light Cycler 480 Probes Master kit (Roche) was used, under the following cycling conditions: 10 min at 95°C, followed by 45 cycles of denaturation (95°C, 10 s), annealing (60°C, 30 s), and extension (72°C, 1 s).

The foreign ribotypes were quantified using SYBR Green I-based assays and the LightCycler 480 SYBR green I master kit (Roche), under the following cycling conditions: 5 min at 95°C, followed by 40 cycles of denaturation (95°C, 10 s), annealing (assay-specific Ta, 10 s; for specific conditions see Supplementary Table 2) and extension (72°C, 15 s). To detect the possible formation of primer dimers or unspecific amplification, the cycling was concluded with a standard melting curve analysis.

The efficiencies of the qPCR assays were estimated from standard calibration curves based on serial 10-fold dilutions of plasmid standards with specific ribotype sequence inserts, ranging from 10^6^ to 1 copies of target nrDNA. The absolute quantification of the target sequences was carried out based on the standard calibration curves using the LightCycler 480 software, version 1.5 (Roche). The resulting concentrations of amplicon DNA, expressed as DNA copy number (ng^–1^), were further normalised to the genome sizes of the *Hordeum* species analysed (Jakob et al., 2004). For each quantification standard, three technical replicates were used, whereas for each analysed individual two technical replicates were included in the qPCR reactions.

### Estimation of nrDNA Copy Number Using Illumina Data

In addition to the qPCR-based estimation of CNV, we estimated CNV of native ribotypes from whole-genome sequencing data. This analysis provides an alternative method to the qPCR-based estimation, enabling estimation of relative sensitivity of both methods. Therefore, only a subset of *Hordeum* samples was included in this analysis (specified below). In principal, the CNV was calculated from the number of Illumina reads mapped to a reference out of the total number of reads used for the mapping. The CN estimation was calculated as described in Wang et al. (2019), with two modifications. First, to avoid potential bias caused by uneven coverage within the rDNA region (genes vs. spacers), we used a complete repeat unit (18S-ITS1-5.8S-ITS2-26S) as a reference. Second, we used 2C DNA content as input data in the formula to conform with the qPCR-based CN estimation. A unique reference was used for each species: Each reference consisted of a species-specific ITS region (retrieved from Mahelka et al., 2017), which was surrounded by universal 18S and 26S rDNA genes. The genes were common to all species and have been derived from *Hordeum bogdanii*. The transcription unit of *H. bogdanii* was completed by mapping genomic reads of *H. bogdanii* against a *Secale cereale* reference (JF489233). The genes’ and spacers’ boundaries were then adjusted with the aid of an alignment consisting of multiple rDNA units of grasses including the newly assembled *H. bogdanii*. Lengths of the reference sequences varied slightly among the *Hordeum* samples (see Table 2). Mapping of reads was done using Bowtie2 (Langmead and Salzberg, 2012) implemented in Geneious version R10 (Biomatters Ltd.), using local alignment type and lowest sensitivity.

**TABLE 2:** Copy numbers of native ribotype in *Hordeum* as estimated using Illumina data.

*Hordeum* sample	BioProject	Experiment accession	Run	Genome size (Mb/2C)	Number of reads (subsample)	Reads mapped	Genome proportion (%)^1^	Genome space (Mb)^2^	Genome space (Kb)	Copy number^3,4^ (rounded)
*H. vulgare* cv. Morex	PRJEB31444^a^	ERX3211415	ERR3183564	10357.02	35000000	178318	0.51	52.781	52781	9100
*H. vulgare* cv. BW457	PRJEB3038	ERX103249	ERR127100	10357.02	34190711	251901	0.74	76.306	76306	13150
*H. vulgare* cv. AAC Synergy	PRJNA665698^b^	SRX9220769	SRR12748507	10357.02	35000000	124428	0.36	36.820	36820	6350
*H. vulgare* cv. Igri	PRJEB36576^c^	ERX4041629	ERR4040343	10357.02	35000000	116486	0.33	34.470	34470	5940
*H. vulgare* subsp. *spontaneum*	PRJEB25923^d^	ERX2779022	ERR2766181	10435.26	35000000	206923	0.59	61.694	61694	10640
*H. murinum* subsp. *glaucum* BCC2017	PRJNA491526^e^	SRX4789710	SRR7956029	8909.58	31000000	158437	0.51	45.536	45536	7850
*H. marinum* BCC2001	PRJNA720259^f^		SRR14162036	8899.8	48000000	112020	0.23	20.770	20770	3580
*H. gussoneanum* BCC2012	PRJNA720259^f^		SRR14162035	10180.98	54086101	71061	0.13	13.376	13376	2310
*H. bogdanii* BCC2063	PRJNA720259^f^		SRR14162034	9271.44	47328373	73152	0.15	14.330	14330	2470
*H. pubiflorum* BCC2028	PRJEB1812^g^	ERX246085	ERR271809	8508.60	44000000	89665	0.20	17.339	17339	2990

*Superscript letters in BioProjects refer to studies in which the data were generated (if available). Genome size values are from Jakob et al. (2004).^1^Calculated as [(number of mapped reads/total number of reads) × 100].^2^Calculated as [(genome proportion × genome size)/100].^3^Calculated as [(genome space in kb/length of the reference rDNA unit in kb].^4^Reference rDNA unit lengths: H. vulgare (incl. ssp. spontaneum), H. bogdanii 5.801 kbp, H. marinum, murinum, gussoneanum 5.803 kbp, H. pubiflorum 5.799 kbp.^a^Monat et al., 2019, ^b^Xu et al., 2021, ^c^Jayakodi et al., 2020, ^d^Liu et al., 2019, ^e^Kono et al., 2019, ^f^this study, ^g^Mascher et al., 2013.*

We used for the analysis samples representing all major *Hordeum* lineages (genomes H, Xu, Xa, and I; Blattner, 2009): *H. vulgare* subsp. *vulgare* (cultivars Morex, BW457, AAC Synergy, Igri; genome H), *H. vulgare* subsp. *spontaneum* (H), *H. murinum* subsp. *glaucum* (Xu), *H. marinum* (Xa), *H. gussoneanum* (Xa), *H. bogdanii* (I), and *H. pubiflorum* (I). For *H. murinum* subsp. *glaucum*, *H. marinum*, *H. gussoneanum*, *H. bogdanii*, and *H. pubiflorum*, we used the same accessions (but not individuals), as we used for the qPCR-based estimation of CN (compare Table 2 and Supplementary Table 1). Short read archives were either downloaded from NCBI database, or we used our unpublished data (Table 2). For each species, the reads were subsampled to ca 1 × genomic coverage. Details on the accessions analysed and short read archives used for the analysis are provided in Table 2.

### Identification of nrDNA Loci Number

The number of nrDNA loci was determined by fluorescent *in situ* hybridisation on metaphase chromosomes using the pTa71 probe as described in Mahelka and Kopecký (2010). The experiments were done under conditions of ∼77% stringency.

### Data Analysis

We carried out a number of basic exploratory statistics to evaluate the variation of estimated nrDNA copy numbers. To determine the effects of variables, we used linear regression, ANOVA, Tukey’s HSD test and two-way ANOVA in R version 3.6.3 (R Core Team, 2020).

## Results

We carried out a qPCR-based estimation of nrDNA copy number in the diploid representatives of the genus *Hordeum*. The main objective was to investigate the dynamics and evolution of the foreign ribotypes, in particular by estimating the CNV of the foreign ribotypes and comparing these with the CNV of native *Hordeum* ribotypes. For a subset of taxa, representing all major *Hordeum* clades (genomes), we performed an alternative estimation of CNV of native ribotype using low coverage Illumina sequencing.

### Copy Number Estimates Based on qPCR and Illumina Read Mapping Are Correlated

The copy numbers of the native ribotype estimated using qPCR and Illumina read mapping differed in all analysed taxa. In all samples, mean qPCR-based values were higher than the values obtained from read mapping. The biggest difference was observed for *H. murinum* (4×), whereas in *H. pubiflorum* the difference was much smaller (1.3×, Supplementary Table 3). Overall, the copy numbers estimated using the two methods were correlated (adjusted *R*^2^ = 0.743, *p* = 0.0078; Supplementary Figure 1). Since we focus primarily on relative copy number differences, and a detailed sample including all diploid *Hordeum* species was used only to estimate CN based on qPCR, we consider qPCR as the default method. Therefore, CN estimates are hereafter qPCR-based, unless otherwise stated.

### Copy Number of Native Ribotype Varies Between Species and Individuals

For the native *Hordeum* ribotype, we found an up to 19-fold difference in the mean copy number among the taxa analysed. The lowest mean value, 1689 copies per 2C content, was estimated for *H. patagonicum* subsp. *mustersii* and the highest, 31,342 copies, for *H. murinum* subsp. *glaucum* (Table 1). A high variation in copy number (up to fourfold) was also observed between different accessions of the same taxa (Supplementary Table 1). To determine whether the observed variation in nrDNA copy number was affected by variations in the DNA extraction efficiency (and therefore represents a methodological artefact), we carried out a regression analysis. We identified no correlation between DNA extract concentration (as a measure of DNA extraction efficiency) and nrDNA copy number (adjusted *R*^2^ = −0.025, *p* = 0.71; Supplementary Figure 2). This indicates that there is no significant effect of DNA extraction efficiency on the measured CN value. For *H. vulgare* subsp. *vulgare*, intraspecific CNV could be estimated also from Illumina read mapping. Here, the four accessions (different cultivars) showed up to a 2.21-fold variation in the copy number (Table 2).

### Phylogeny, Genome Size, and Number of nrDNA Loci Have No Effect on CNV of the Native Ribotype

**FIGURE 1:**
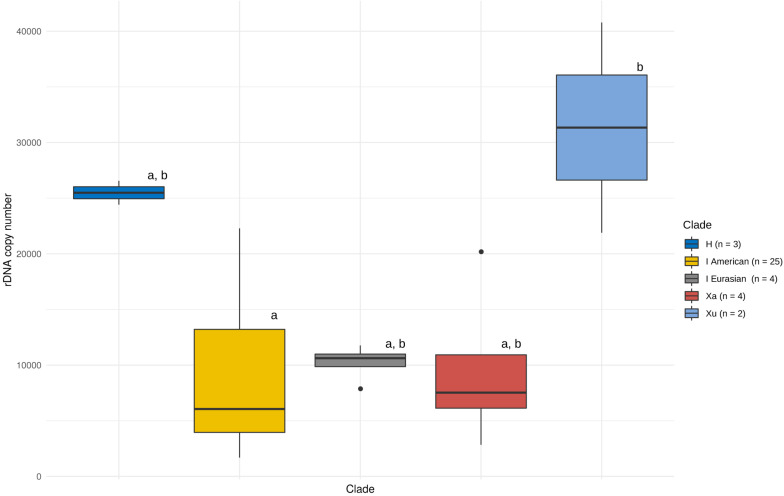
Copy number variation of the native ribotype in *Hordeum*. Variation within and between major phylogenetic groups is shown. The boxes represent the range between 25th and 75th percentil. The whiskers represent the distance between hinges to the 1.5 × IQR (IQR is the inter-quartile range, or distance between the first and third quartiles) to both sides (toward the maximum as well as minimum values). Vertical bars represent the median values and outlayers are dispalyed as individual points. Phylogenetic grouping is based on Brassac and Blattner (2015). Differences among means (solid lines in boxes) at *P* = 0.05 (as analysed by ANOVA) are indicated by letters above boxes (boxes with the same letters are not significantly different).

We assessed the effect of genome size, number of nrDNA loci and phylogeny on the observed patterns of CNV. There were either one or two pairs of nrDNA loci in the *Hordeum* samples analysed (Supplementary Table 1 and Supplementary Figures 3, 4). We found no significant relationship between the copy number of the native ribotype and genome size or the number of nrDNA loci (Supplementary Figure 5). Conversely, the nrDNA copy number was significantly affected by a group’s phylogeny (ANOVA, *F* = 3.396, *p* < 0.05). However, the *post hoc* comparisons (Tukey’s HSD test) revealed that the only significant difference was that between the Xu-genome and the American I-genome species (Figure 1).

The CNV was high within the recognised phylogenetic groups (Table 3). The highest variation (in the ratio of maximal and minimal values, estimated within a lineage) was observed among the American I-genome species (13.2-fold). On the other hand, the H genome, represented by *H. vulgare*, was the most homogeneous group with respect to nrDNA copy number. Within this group, the two subspecies differed only by a factor of 1.1 (Table 3).

**TABLE 3:** Copy number variation of native *Hordeum* ribotype within the major phylogenetic lineages.

Lineage	Mean copy number (s.d.)	Copy number variation
H	25485 (1513)	1.1
I – American	8682 (6392)	13.2
I – Eurasian	10229 (1636)	1.5
Xa	9528 (7470)	7.1
Xu	31342 (13353)	1.9

*Phylogenetic lineages follow the phylogeny of Brassac and Blattner (2015); standard deviation (brackets); copy number variation – ratio of maximal and minimal values estimated within each lineage.*

### Foreign Ribotypes Represent a Minor Proportion of Total nrDNA

**FIGURE 2:**
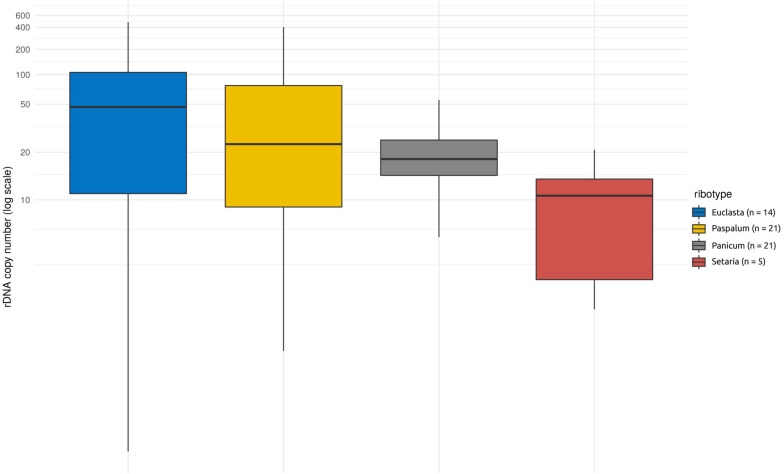
Copy number variation within and among the foreign ribotypes in *Hordeum* species of sect. *Stenostachys*. No significant differences at *P* = 0.05 were detected using ANOVA.

Apart from the native nrDNA, the I-genome *Hordeum* species contain foreign ribotypes acquired from panicoid grasses. The sequence divergence of particular ribotypes allowed us to design specific qPCR assays targeting four out of the five foreign ribotypes (namely *Panicum*-, *Setaria*-, *Paspalum*-, and *Euclasta*-like) and hence to estimate their CNV. The estimated copy number of the foreign ribotypes was remarkably low compared with that of the native ribotypes. The estimates ranged from a few copies to tens, solely hundreds of copies (Table 1). Individual foreign ribotypes represent only a small fraction of the estimated total number of nrDNA copies (0.01 – 12.02% depending on ribotype; Supplementary Table 1). Overall, the *Euclasta*-like ribotype was the most abundant and the *Setaria*-like ribotype was the least abundant (Figure 2). The two remaining ribotypes showed intermediate values. The differences among the abundances of the foreign ribotypes were not significant (ANOVA, *F* = 2.572, *p* = 0.06).

We carried out a regression analysis to test whether the copy numbers of the foreign ribotypes were affected by the copy number of the native ribotype. We found the estimated abundances of foreign ribotypes were independent of the abundance of the native ribotype (Supplementary Figure 6). This indicates the high specificity of the qPCR assays (indicating the absence of significant cross-amplification of the non-target template by the qPCR assays). It points to the independent evolution of the foreign and native ribotypes within the genomes studied.

### Phylogeny and Geography Have No Effect on the Copy Numbers of Foreign Ribotypes

**FIGURE 3:**
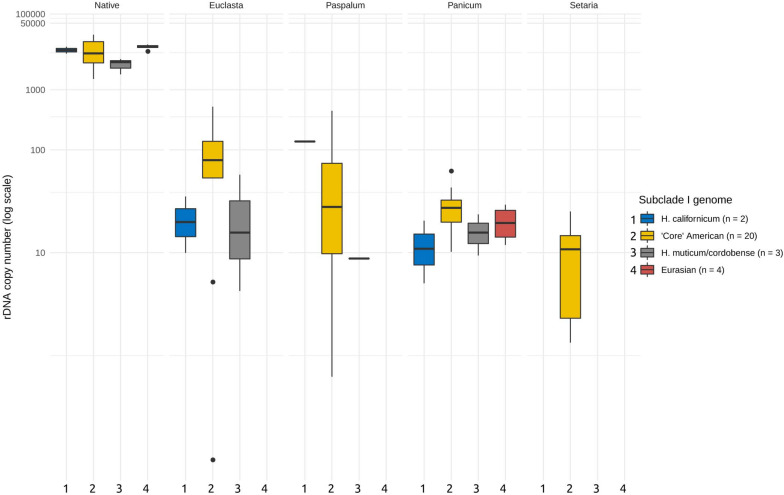
Copy number variation of the foreign ribotypes in relation to major phylogenetic groups. Phylogenetic grouping is based on Brassac and Blattner (2015). Foreign ribotypes only occur in *Hordeum* species of sect. *Stenostachys*. No significant differences at *P* = 0.05 were revealed by ANOVA. Copy numbers of native ribotype are given for comparison.

Next, we asked whether the abundances of the foreign ribotypes correlated with the phylogenetic relationships of the I-genome *Hordeum* species. For this purpose, we subdivided the I-genome group into subclades, which reflected the group’s phylogeny (Table 1). We found no significant relationship between the abundance of the foreign ribotypes and the phylogeny (ANOVA, *F* = 0.758, *p* = 0.52 for the differences between the subclades, *F* = 0.968, *p* = 0.43 for the interaction of the subclade and ribotype, Figure 3). We further investigated whether the CNV of the foreign ribotypes was affected by the geographic origins of the species analysed. Again, the copy number of the foreign ribotypes did not differ significantly among samples of different geographic origin (ANOVA, *F* = 1.077, *p* = 0.37) and the interactions between the foreign ribotype and geographic origin of the sample were not significant either (*F* = 0.551, *p* = 0.74).

## Discussion

Apart from their native nrDNA copies, species of section *Stenostachys* from the barley genus *Hordeum* harbour foreign nrDNA copies, presumably acquired after a series of HGT events from panicoid grasses (Mahelka et al., 2017). To investigate the evolution and dynamics of the foreign ribotypes, we estimated their CNV using specific qPCR assays, and related the values to the CNV of native *Hordeum* ribotypes. We hypothesised that the HGTs resulted in insertions of foreign nrDNA arrays at random places across *Hordeum* genomes, so that the foreign ribotypes represent non-functional entities, which reside apart from the NORs in the *Hordeum* species. Such a pattern would imply independent evolution of native and foreign ribotypes, which can be tested using relationships between CNV of foreign and native ribotypes.

### The Usefulness of qPCR and Mapping of Short Reads for Quantification of nrDNA

To investigate the differences in CNV of individual ribotypes, we targeted our qPCR assays to ITS of nrDNA. These regions showed sufficient variation to discriminate between the foreign ribotypes, as well as between the foreign ribotypes and the native one. One of the assays was used to estimate the CNV of the native (*Hordeum*) ribotype across the genus *Hordeum*. We used this method mainly to seek for relative differences in CNV of particular ribotypes in the different species, or in the major phylognetic groups. For precise estimates of absolute copy numbers, one should target the rRNA genes (Long et al., 2013; Wang et al., 2019), ideally coupled with a normalisation using a well-defined and confirmed single-copy gene(s) (Long et al., 2013; Rosato et al., 2016; Rabanal et al., 2017). Conversely, specific qPCR assays targeting the ITS region, capture all target sequences irrespective of their genomic locations, and thus represent an efficient tool for detection of potentially pseudogenised, interspersed, non-native nrDNA copies. Thus, this approach is suitable for detection and quantification of minority acquired copies, for example via HGT.

To get insight into the sensitivity of the qPCR-based CN estimation, we implemented an alternative method based on Illumina read mapping. We thus obtained an independent estimate of CNV for a subset of taxa, representing all the major phylogenetic groups included in the study. We found considerable differences between the two methods, with qPCR-based CN estimates showing 1.3–4 × higher values (Supplementary Table 3). Despite the differences between the absolute values, the overall CN estimates were correlated. Hence, we believe that relative differences (between taxa and ribotypes), as inferred from qPCR here, are valid.

The consistently higher CN values estimated using qPCR, than those estimated by read mapping, may partly stem from different methodology of both approaches. While the qPCR targeted ITS region and therefore measured CN thereof, using Illumina data we estimated the number of complete rDNA units. It is likely that the ITS region outnumbers complete rDNA repeat units in plant genomes, because additive ITS copies are scattered across genomic locations outside the main NORs. We found that there are tens of ITS1 copies dispersed across all chromosomes except chr3 within the Morex V2 assembly of *H. vulgare* (Monat et al., 2019) (Supplementary File 1). In *Hordeum* samples analysed by us, a visual inspection of mapped reads suggests that there is no obvious increase in coverage within the ITS region. On the other hand, there is a decrease of reads, which mapped to the 26S gene in *H. bogdanii*, *H. marinum*, and *H. vulgare* cultivars BW457 and AAC Synergy. Thus, Illumina-based CNs can be underestimated in these cases. In any case, uneven coverage of a region of interest by reads is a potential source of error in CN estimation based on read mapping.

Potential discrepancies of relative values (e.g., the opposite ratios of CNs obtained for *H. bogdanii* accession BCC2063 and *H. pubiflorum* BCC2028; Table 2 and Supplementary Table 1), can be attributed to intraspecific variation of CN, when differences among individuals are commonplace (discussed below). In this particular case (*H. bogdanii* vs. *H. pubiflorum*), identical accessions were used, but not the same individuals. Even the use of plant material from a seed bank is not a guarantee of genotype homogeneity. For example, Jakob et al. (2014) studied genetic diversity in wild barley *Hordeum vulgare* subsp. *spontaneum*. Interestingly, *ex situ* samples of this taxon showed genetic admixture, as 22% of gene bank samples did not correspond with the geographic pattern found in wild barley accessions collected in the field (in which the incongruence was only found in 5% of samples). The authors speculated, that although *H. vulgare* is classified as an inbreeder, the admixture was likely caused by hybridisation during propagation and maintenance, and, possibly, handling of the *ex situ* gene bank samples. Thus, different individuals may actually represent diverse genotypes even if they are maintained under the same accession number. In this respect, revealing the net effect of particular methods investigated in the same individual plants (or even better in the same DNA extract), would be necessary.

### CNV Among Ribotypes

One result of the multiple HGTs from panicoid grasses into *Hordeum* is that the I-genome species (and even some individual plants) harbour up to five foreign ribotypes. Moreover, these correspond to distinct panicoid genera, namely *Panicum*, *Paspalum*, *Setaria*, *Euclasta*, and *Arundinella* (Mahelka et al., 2017). In most cases, multiple foreign ribotypes were found in single individual plants. One individual of *H. stenostachys* even combined foreign ribotypes from all of the five panicoid genera. Although the distribution of foreign ribotypes among the I-genome species has been well described, information on their quantity, dynamics (variation among individuals) and evolution (variation among species) is largely lacking.

The CNV of individual foreign ribotypes provides valuable information, as it may indicate potential functionality and thus evolutionary potential in *Hordeum* genomes through their quantity and inferred chromosomal localisation. In another study, Mahelka et al. (2021) provide detailed characterisation of the *Panicum*-derived chromosomal segment in *Hordeum* species. The authors reconstructed the foreign DNA segment using sequencing of BAC clones in *H. bogdanii* (specimen BCC2063) and *H. pubiflorum* (BCC2028). There were two *Panicum*-like copies of the ITS region identified within the segment, and the copies occurred at two distinct sites. Thus, given the confirmed presence of a *Panicum*-derived segment on both chromosomal homologs, there are at least four *Panicum*-like copies in *H. bogdanii* and *H. pubiflorum*. The copy number of *Panicum*-like ribotypes as inferred from qPCR shows higher values (21 copies in *H. bogdanii* BCC2023 and 29 copies in *H. pubiflorum* BCC2028). This higher count can be explained by the presence of additional copies beyond the boundaries of the *Panicum*-derived segment, which would not have been detected by Mahelka et al. (2021). Furthermore, we still cannot rule out the possibility that some of the foreign ribotypes reside within the native nrDNA loci. The coexistence of different nrDNA variants within a single genome has been documented in allopolyploid and homoploid hybrids (e.g., Kovařík et al., 2008; Fehrer et al., 2009; Zozomová-Lihová et al., 2014) or even in presumed pure diploid species with different paralogous rDNA loci (Blattner, 2004). For these, several scenarios have been described, including maintenance of the different ribotypes within their original loci, formation of new recombinant ribotypes or gradual replacement of one ribotype by another due to interlocus recombination (reviewed in Álvarez and Wendel, 2003). We found only a small proportion of nrDNA is of foreign origin, supporting their presence solely in minor arrays, co-localised with other genetic material of foreign origin as expected under the HGT scenario (Mahelka et al., 2017, 2021). Due to their localisation, out of reach of homogenisation mechanisms, such minor arrays may remain conserved in plant genomes in relatively stable amounts over millions of years (Mahelka et al., 2021).

### CNV of nrDNA Is High Both Within and Between Species, and Is Independent of Phylogeny, Genome Size, and Number of nrDNA Loci in *Hordeum* Species

A high level of CNV of the native ribotype detected between individuals of the same species (e.g., a 4-fold difference in *H. intercedens*) as well as between species within phylogenetic groups (e.g., a 13.2-fold difference within the American I-genome species) is not unusual. In other studies, high variation in nrDNA copies has been reported not only among closely related species and among individuals within a species but even between different tissues of the same individual. For example, Govindaraju and Cullis (1992) detected a 12-fold variation in nrDNA copy numbers among individuals within populations of *Pinus rigida*, whereas variation between populations reached values up to 21-fold. Populations of *Arabidopsis thaliana* from northern and southern Sweden differed in their genome size by more than 10%, mainly due to the variability in nrDNA copy number (Long et al., 2013). In *Vicia faba*, nrDNA copy number was shown to differ between individuals within a population (up to 95-fold differences) and between different tissues of the same individual, showing up to a 12-fold difference (Rogers and Bendich, 1987). Mechanisms underlying such differences still remain to be revealed. In *Hordeum*, the highest variation within phylogenetic groups was observed for the American I-genome species. In this case, the results may have been partly skewed by the number of samples, since the I-genome group is the most species-rich within *Hordeum*. Since we aimed to analyse two individuals per each species, some of the species-poor clades (H, Xu) were represented by only two individuals. Multiple samples from within other than I-genome group were only included for *H. vulgare* (H genome), in which CN of four cultivars was estimated using read mapping (Table 2). These four samples showed variation that was twice that of the two samples from the H group estimated using qPCR (*H. vulgare* subsp. *vulgare* and *spontaneum*). Since intra-specific variation of rDNA was described for both (Saghai-Maroof et al., 1984), it is likely that the inclusion of more wild barley individuals could further increase the CNV within the H-genome group.

In *Hordeum*, genome size is correlated with the genus’ phylogeny (Jakob et al., 2004). Our data on the CNV of nrDNA suggest that this correlation is not due to quantity of nrDNA. Despite the high level of CNV detected between *Hordeum* species, the only significant difference was that between the Xu- and the American I-genome species. We did not find a significant correlation between the nrDNA copy number and the genome size even when individual plants were examined (Supplementary Figure 5A). We further questioned whether the observed CNV could be an effect of the number of nrDNA loci. Since the number of nrDNA loci (particularly of the minor ones) varies in *Hordeum* (Taketa et al., 1999, 2001), using nrDNA-FISH we identified the number of loci in the same accessions, as used for the CNV analysis. The *Hordeum* species involved in this study carried one or two pairs of loci (Table 1). This is in accord with other studies, reporting for diploid species one or two pairs of major loci plus up to four pairs of minor loci (Leitch and Heslop-Harrison, 1992; Taketa et al., 1999, 2001). The absence of a significant relationship between CNV and the number of nrDNA loci suggests that the number of loci is not responsible for the variation in nrDNA copy number in *Hordeum*. Instead, within-loci dynamics (causing contraction or expansion of nrDNA arrays) seems to be the dominant mechanism underlying CNV in this group. A striking example fitting this hypothesis is *H. murinum* subsp. *glaucum* analysed here. The hybridisation signal after nrDNA-FISH in accession BCC2017 (Supplementary Figure 4B) is particularly stronger than that observed in the other accession of the same species (BCC2002, Supplementary Figure 4A). This pattern is consistent with copy number of nrDNA, when accession BCC2017 contains almost twice the number of copies. Although nrDNA-FISH is not a precise method to quantify copy number of rDNA, a correlation between copy number of nrDNA and intensity of hybridisation signal presumably exists.

At inter-specific level, the relationship between nrDNA copy number and phylogeny is still poorly explored. Inter-specific genome size evolution has been addressed for almost two decades, especially for plants (e.g., Albach and Greilhuber, 2004; Jakob et al., 2004; Weiss-Schneeweiss et al., 2006; Chrtek et al., 2009; Kang et al., 2014; Mandák et al., 2016). Besides investigating the patterns of genome size variation in a phylogenetic context, recent studies have focused on identification of particular genomic components as the main drivers of these changes (Zedek et al., 2010; Talla et al., 2017; Blommaert et al., 2019; Hloušková et al., 2019; Wong et al., 2019; McCann et al., 2020; Vitales et al., 2020). To date, nrDNA has been confirmed as being a substantial driver of interspecific genome size evolution only in a group of ground beetles (Sproul et al., 2020). Otherwise, no obvious relationship has been observed to enable generalisation of nrDNA evolution.

## Conclusion

Here, we reveal the patterns of copy number variation of nrDNA in diploid species of the genus *Hordeum*. For this purpose, we categorised the nrDNA types as native or foreign ribotypes. While the native, *Hordeum*-like, ribotype is present in all *Hordeum* species, the occurrence of foreign ribotypes is restricted to the I-genome *Hordeum* species (*Hordeum* sect. *Stenostachys*), which acquired these ribotypes via a series of horizontal transfers. The foreign ribotypes were present in the respective genomes only at low copy numbers (a few copies to hundreds of copies) and represent a relatively minor fraction of the total nrDNA. We detected a high level of variation in the copy numbers of particular ribotypes at all hierarchical levels examined – between species groups, between species, and between individuals within a species. This variation did not correspond to any of the genus’ phylogeny, the species’ genome size, or the number of nrDNA loci. Overall, we can consider nrDNA copy number as a dynamic trait in *Hordeum*.

## Data Availability Statement

The datasets presented in this study can be found in online repositories. The names of the repository/repositories and accession number(s) can be found in the article/Supplementary Material.

## Author Contributions

KK, VM, and PC: conceived and designed the study. PC: conducted lab experiments. DK: provided *in situ* hybridisation experiments. FB: provided the Illumina data. KK and VM: analysed the data. KK: drafted the manuscript with contribution from VM. All co-authors contributed to the final version of the manuscript.

## Conflict of Interest

The authors declare that the research was conducted in the absence of any commercial or financial relationships that could be construed as a potential conflict of interest.

## Footnotes

1 http://cels.uri.edu/gsc/cndna.html

## Supplementary Material

Supplementary Figure 1 Correlation between copy numbers of native ribotype estimated using qPCR and Illumina read mapping.

Supplementary Figure 2 Correlation between the DNA extraction efficiency (expressed as DNA extract concentration) and the copy number of nrDNA, for both native **(A)** and foreign **(B)** ribotypes.

Supplementary Figure 3 Molecular cytogenetic analysis of *Hordeum* species. Fluorescent signal of 45S-rDNA (labeled with biotin, arrows) has been observed after FISH on one or two pairs of chromosomes of *H. bogdanii* BCC2063 **(A)**, *H. bulbosum* GRA970 **(B)**, *H. californicum* BCC2057 **(C)**, *H. cordobens*e BCC2067 **(D)**, *H. erectofolium* BCC2026 **(E)**, *H. euclaston* BCC2022 **(F)**, *H. flexuosum* BCC2023 **(G)**, *H. intercedens* BCC2044 **(H)**, and *H. marinum* BCC2001 **(I)**. In *H*. *cordobense* **(D)** and *H. erectifolium* **(E)**, minor loci of 45S-rDNA on one pair of chromosomes have been observed (arrowheads). *Hordeum bulbosum* GRA970 is tetraploid (2*n* = 28), hence it was excluded from qPCR analyses. Scale bar 10 μm.

Supplementary Figure 4 Molecular cytogenetic analysis of *Hordeum* species. Fluorescent signal of 45S-rDNA (labeled with biotin, arrows) has been observed after FISH on one or two pairs of chromosomes of *H. murinum* BCC2002 **(A)**, *H. murinum* BCC2017 **(B)**, *H. muticum* BCC2042 **(C)**, *H. patagonicum* subsp. *setifolium* BCC2032 **(D)**, *H. pubiflorum* BCC2028 **(E)**, *H. pusillum* BCC2049 **(F)**, *H. roshevitzii* BCC2015 **(G)**, *H. stenostachys* BCC2041 **(H)**, and *H. vulgare* cv. Morex **(I)**. In *H. murinum* BCC2017 **(B)** and *H. vulgare* cv. Morex **(I)**, minor loci of 45S-rDNA on one pair of chromosomes have been observed (arrowheads). Scale bar 10 μm.

Supplementary Figure 5 **(A)** Correlation between the genome size (2C value) and the copy number of native ribotype, **(B)** correlation between the number of nrDNA loci and the copy number of native ribotype.

Supplementary Figure 6 Correlations between the copy numbers of native and foreign ribotypes. **(A)** *Setaria*-like ribotype; **(B)** *Paspalum*-like ribotype; **(C)** *Panicum*-like ribotype; **(D)** *Euclasta*-like ribotype.

Supplementary Table 1 Characteristics of *Hordeum* samples used in the study.

Supplementary Table 2 Detailed description of the qPCR assays used to quantify nrDNA in *Hordeum*.

Supplementary Table 3 Comparison of copy numbers of native ribotype estimated using qPCR and Illumina read mapping.

Supplementary File 1 The distribution of internal transcribed spacer 1 (ITS1) of nuclear ribosomal DNA in the genome of *Hordeum vulgare*.
