# Supplementary figures and images for: Horizontally Acquired nrDNAs Persist in Low Amounts in Host Hordeum Genomes and Evolve Independently of Native nrDNA
Source: Front Plant Sci. 2021 May 17;12:672879. doi: 10.3389/fpls.2021.672879 (PMC8165317; doi:10.3389/fpls.2021.672879)

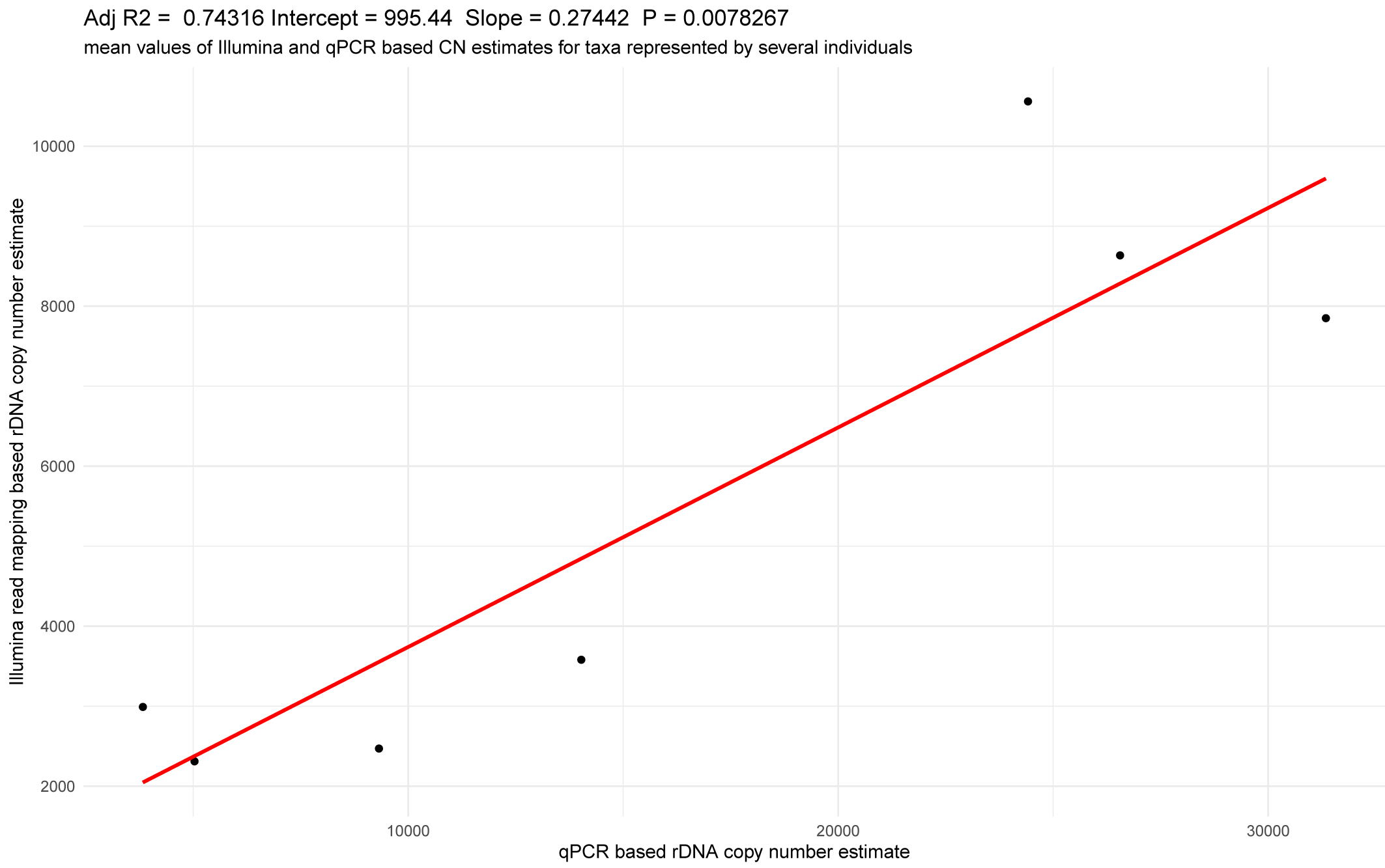

Supplement: Supplementary Figure 1 — Correlation between copy numbers of native ribotype estimated using qPCR and Illumina read mapping. [file Image_1.JPEG]

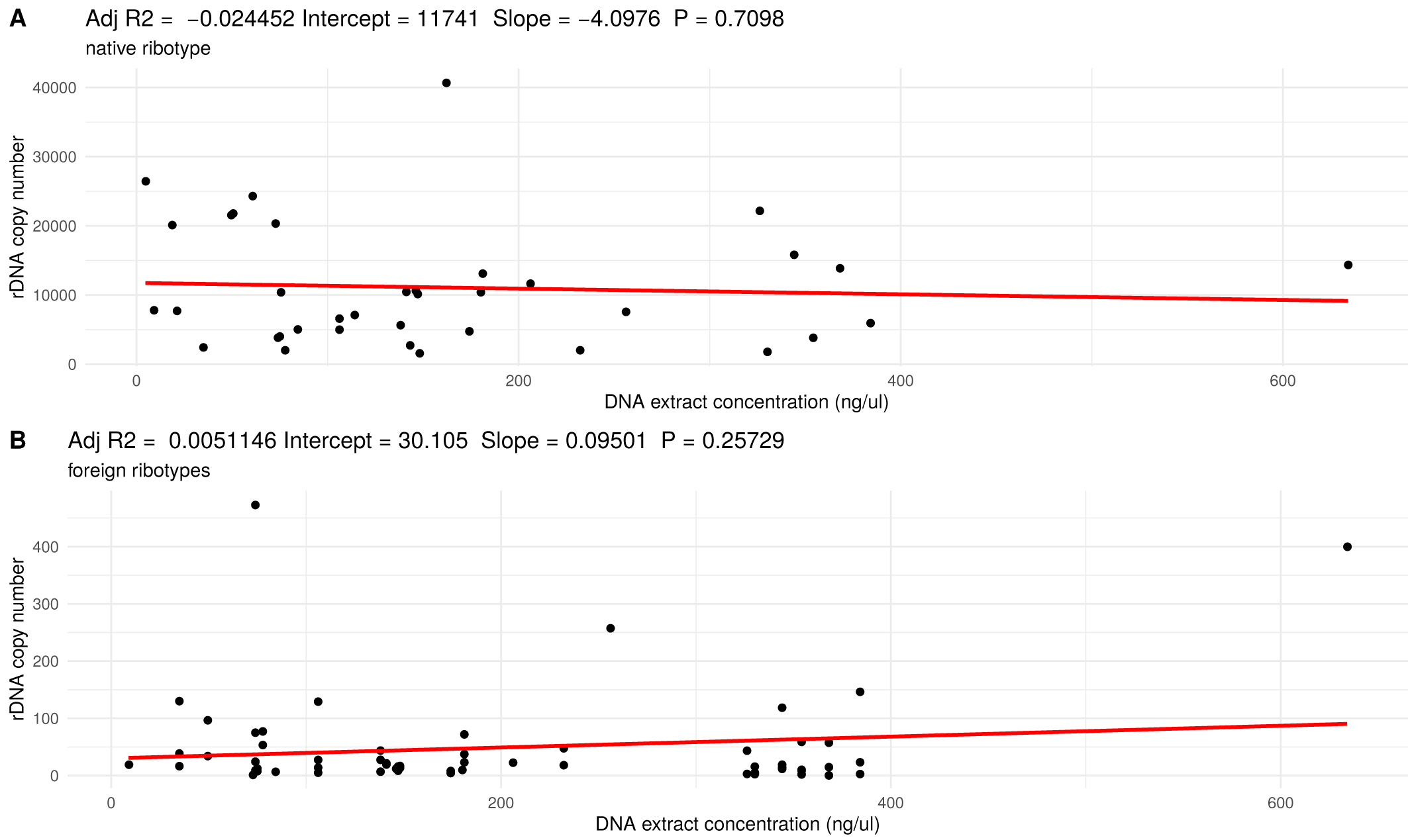

Supplement: Supplementary Figure 2 — Correlation between the DNA extraction efficiency (expressed as DNA extract concentration) and the copy number of nrDNA, for both native (A) and foreign (B) ribotypes. [file Image_2.JPEG]

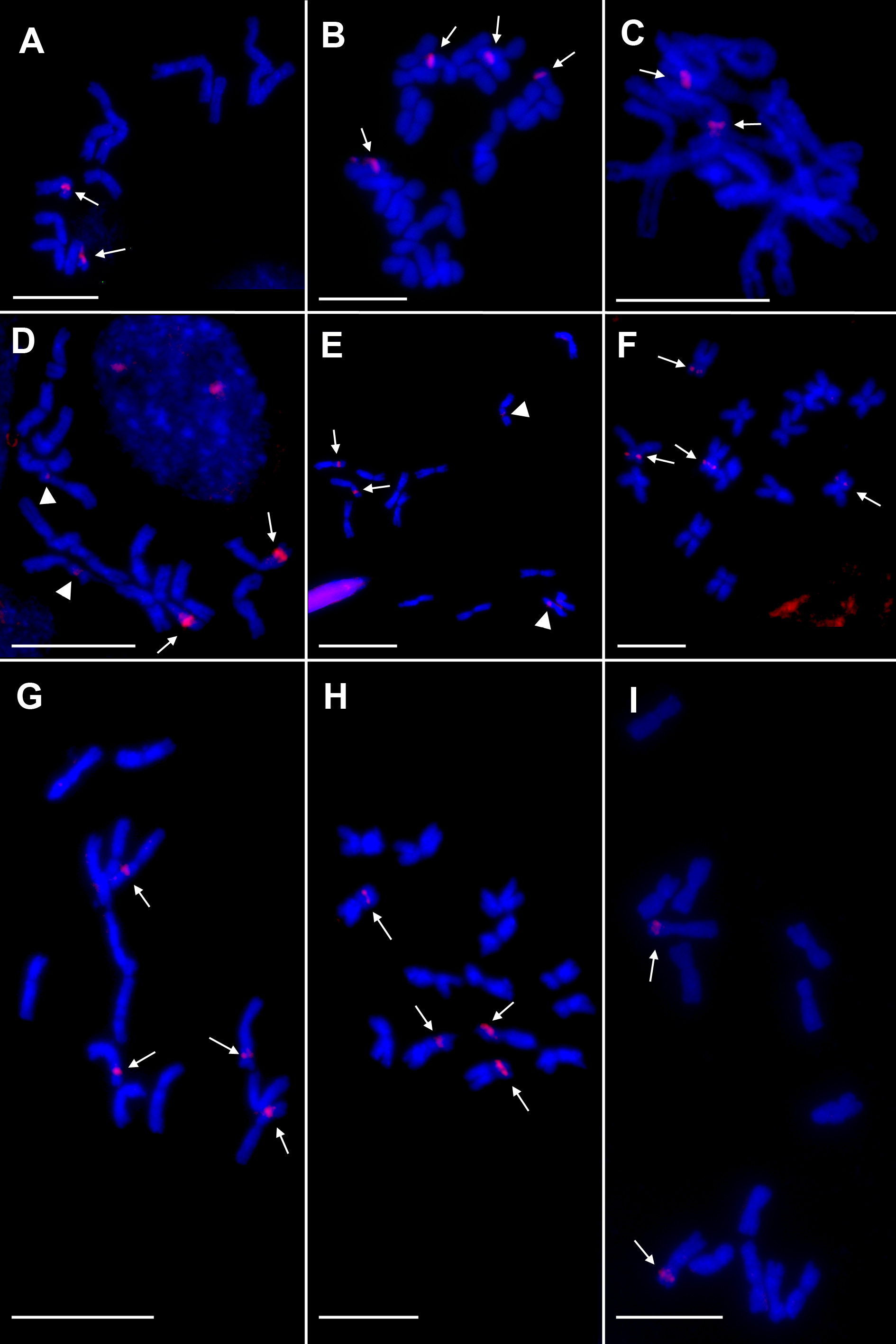

Supplement: Supplementary Figure 3 — Molecular cytogenetic analysis of Hordeum species. Fluorescent signal of 45S-rDNA (labeled with biotin, arrows) has been observed after FISH on one or two pairs of chromosomes of H. bogdanii BCC2063 (A), H. bulbosum GRA970 (B), H. californicum BCC2057 (C), H. cordobense BCC2067 (D), H. erectofolium BCC2026 (E), H. euclaston BCC2022 (F), H. flexuosum BCC2023 (G), H. intercedens BCC2044 (H), and H. marinum BCC2001 (I). In H. cordobense (D) and H. erectifolium (E), minor loci of 45S-rDNA on one pair of chromosomes have been observed (arrowheads). Hordeum bulbosum GRA970 is tetraploid (2n = 28), hence it was excluded from qPCR analyses. Scale bar 10 μm. [file Image_3.JPEG]

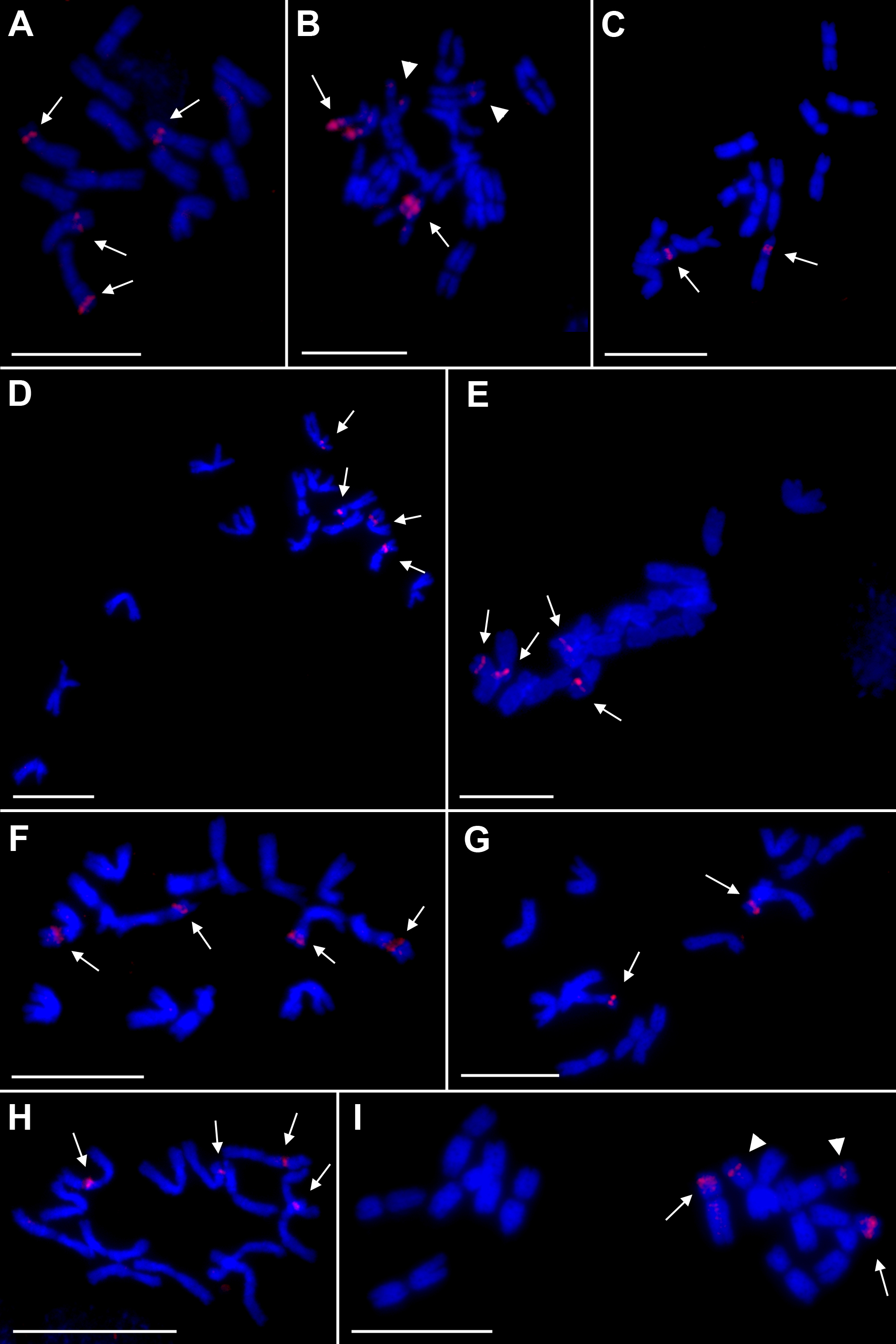

Supplement: Supplementary Figure 4 — Molecular cytogenetic analysis of Hordeum species. Fluorescent signal of 45S-rDNA (labeled with biotin, arrows) has been observed after FISH on one or two pairs of chromosomes of H. murinum BCC2002 (A), H. murinum BCC2017 (B), H. muticum BCC2042 (C), H. patagonicum subsp. setifolium BCC2032 (D), H. pubiflorum BCC2028 (E), H. pusillum BCC2049 (F), H. roshevitzii BCC2015 (G), H. stenostachys BCC2041 (H), and H. vulgare cv. Morex (I). In H. murinum BCC2017 (B) and H. vulgare cv. Morex (I), minor loci of 45S-rDNA on one pair of chromosomes have been observed (arrowheads). Scale bar 10 μm. [file Image_4.JPEG]

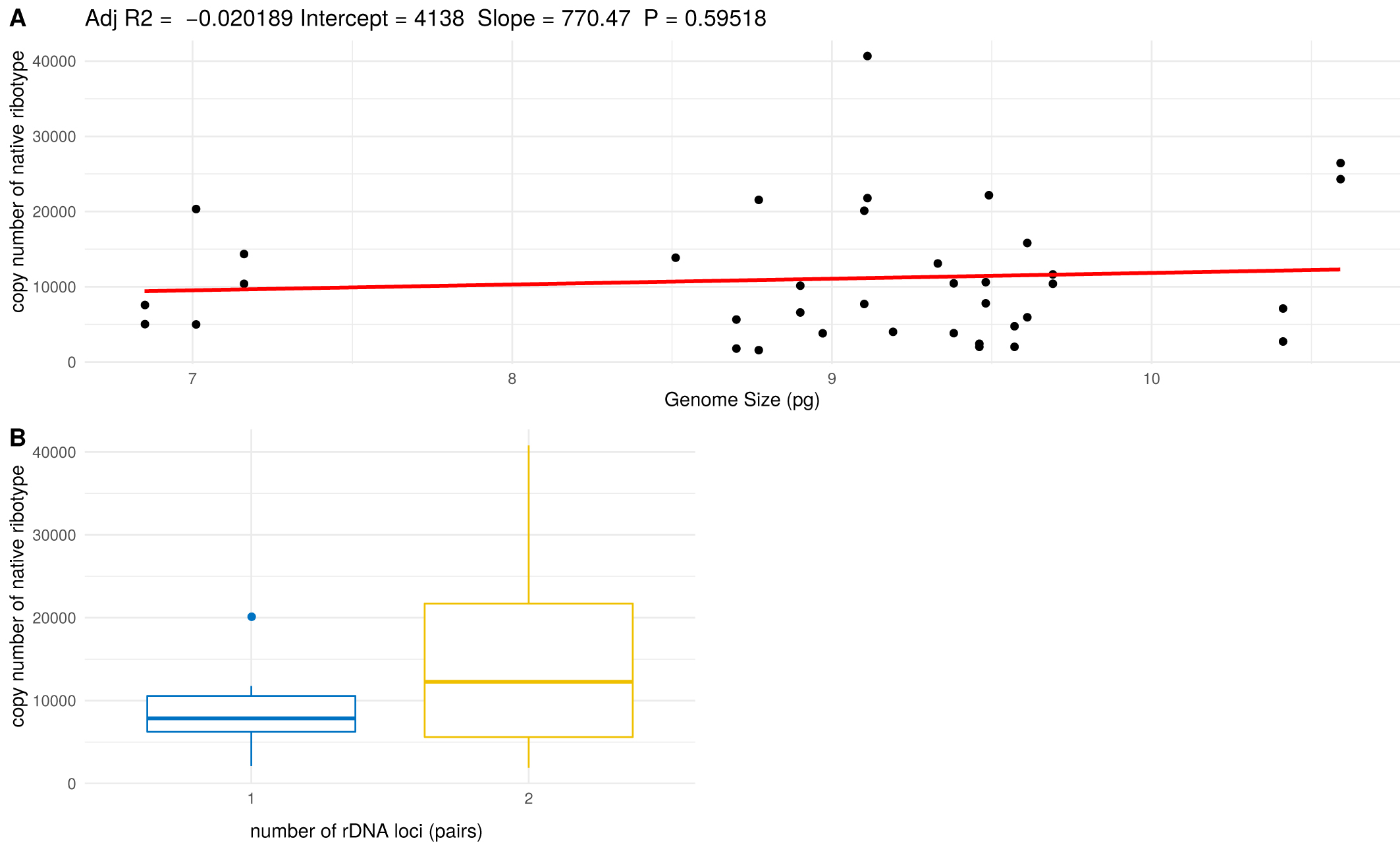

Supplement: Supplementary Figure 5 — (A) Correlation between the genome size (2C value) and the copy number of native ribotype, (B) correlation between the number of nrDNA loci and the copy number of native ribotype. [file Image_5.JPEG]

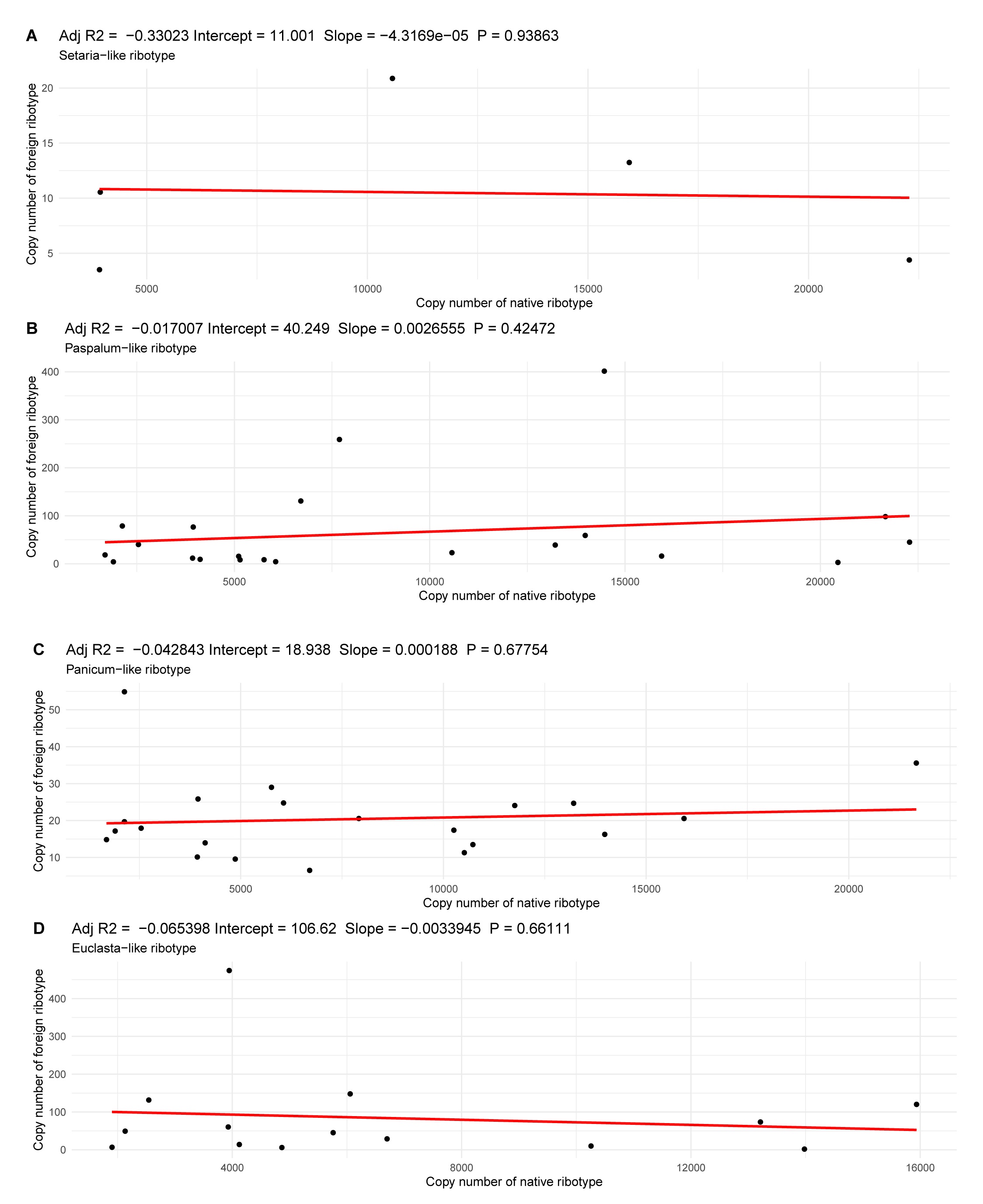

Supplement: Supplementary Figure 6 — Correlations between the copy numbers of native and foreign ribotypes. (A) Setaria-like ribotype; (B) Paspalum-like ribotype; (C) Panicum-like ribotype; (D) Euclasta-like ribotype. [file Image_6.jpg]
