# Supplementary material for: Horizontally Acquired nrDNAs Persist in Low Amounts in Host Hordeum Genomes and Evolve Independently of Native nrDNA
Source: Front Plant Sci. 2021 May 17;12:672879. doi: 10.3389/fpls.2021.672879 (PMC8165317; doi:10.3389/fpls.2021.672879)
Supplement: Supplementary Table 2 — Detailed description of the qPCR assays used to quantify nrDNA in Hordeum. [file Table_2.docx]

| Target ribotype | Assay type | Primers/Probe | Sequence | Concentration (µM) | Ta (°C) |
| --- | --- | --- | --- | --- | --- |
| Native | TaqMan | ALL-222F  ALL-330R  ALL-Probe243b | CACACGACTCTCGGCAAC  TTCAAAGACTCGATGGTTCG  T+A+T+C+TCG+G+C+T+CT | 0.4  0.4  0.2 | 60 |
| *Euclasta* | SYBR Green | Eu102F  Eu481R | ACAGAACCCACGACGCCTT  TGCTGTGCCCGATACGATTC | 0.5  0.5 | 55 |
| *Panicum* | SYBR Green | Pan86F  Pan173R | CGTCAAGGAACACTGATATTG  CATGTGGATTAAGATAGCAACA | 0.5  0.5 | 55 |
| *Paspalum* | SYBR Green | Pas141F  Pas221R | ATATTGCCTTGCGAGGGTGA  GCCGAGAGTCGTGTGGATTA | 0.5  0.5 | 55 |
| *Setaria* | SYBR Green | Set31F  Set138R | CGTGTCATCCATGCCGCAT  ATGTTCCTTGACGCCTTCGG | 0.4  0.4 | 61 |

**Supplementary Table S2.** Detailed description of the qPCR assays used to quantify rDNA ribotypes in *Hordeum*. Concentration refers to the concentration of the primer/probe at the reaction. Ta = annealing temperature. The TaqMan probe for detection of the native ribotype was fluorescently labelled by 6-FAM at the 5’ end in combination with the BHQ quencher at the 3’ end. LNA probe analogues used to increase the Tm of the probe are marked with ‘+’.
