# Supplementary material for: Horizontally Acquired nrDNAs Persist in Low Amounts in Host Hordeum Genomes and Evolve Independently of Native nrDNA
Source: Front Plant Sci. 2021 May 17;12:672879. doi: 10.3389/fpls.2021.672879 (PMC8165317; doi:10.3389/fpls.2021.672879)
Supplement: Supplementary Table 3 — Comparison of copy numbers of native ribotype estimated using qPCR and Illumina read mapping. [file Table_3.docx]

| Taxon | Illumina-based CN | qPCR-based CN | ratio (qPCR/Illumina) |
| --- | --- | --- | --- |
| *H. bogdanii* | 2470 | 9320 | 3.8 |
| *H. gussoneanum* | 2310 | 5031 | 2.2 |
| *H. marinum* | 3580 | 14026 | 3.9 |
| *H. murinum* subsp. *glaucum* | 7850 | 31342 | 4 |
| *H. pubiflorum* | 2990 | 3830 | 1.3 |
| *H. vulgare* subsp. *spontaneum* | 10560 | 24415 | 2.3 |
| *H. vulgare* subsp. *vulgare* | 8635 | 26556 | 3.1 |

**Supplementary Table S3.** Comparison of copy numbers (CN) of native ribotype of nrDNA, estimated using qPCR and Illumina read mapping. Illumina-based CN for *Hordeum* *vulgare* subsp. *vulgare* is the mean value from four cultivars (listed in Table 2). For other samples, Illumina-based CNs are based on single samples. The qPCR-based values are the mean values as given in Table 1.
