# Supplementary material for: Horizontally Acquired nrDNAs Persist in Low Amounts in Host Hordeum Genomes and Evolve Independently of Native nrDNA
Source: Front Plant Sci. 2021 May 17;12:672879. doi: 10.3389/fpls.2021.672879 (PMC8165317; doi:10.3389/fpls.2021.672879)
Supplement: Supplementary File 1 — The distribution of internal transcribed spacer 1 (ITS1) of nuclear ribosomal DNA in the genome of Hordeum vulgare. [file Data_Sheet_1.PDF]

Supplementary file S1. The distribution of internal transcribed spacer 1 (ITS1) of nuclear ribosomal DNA in the genome of *Hordeum vulgare*.

The distribution of ITS1 copies on *H. vulgare* chromosomes was analysed using a combination of BLAST search and sequence mapping. First, we using BLAST identified the positions of ITS1 within *H. vulgare* chromosomes. We used the V2 assembly of *H. vulgare* genome (Monat et al. 2019), from which a BLAST database was built using Geneious 10.2.6. The ITS1 sequence of *H. vulgare* subsp. *spontaneum* (accession F2097; genbank accession number KU513496) was used as the query and BLASTed against the *H. vulgare* genome using Geneious. The retrieved hits were tabulated and sorted according to their position in particular chromosomes. Next, *H. vulgare* chromosomes [including the unanchored sequences (chrUn)] were annotated in Geneious (with 80% similarity) using the sequences of 18S, 26S, 5.8S, and ITS1 and ITS2 (for the genes, we used the sequences of *H. bogdanii*; see main text for details on the rDNA reference) to determine the integrity of the rDNA around the ITS1 regions obtained from the BLAST search. We distinguished between ITS1 located within repeated arrays composed of complete rDNA units, and those located outside these arrays (Table). In general, both the high copy number and repeat size of rDNA pose a serious obstacle in estimating the copy number of rDNA even in high quality genomic assemblies (Nelson et al. 2019). Therefore, the results presented here should not be interpreted as an estimate of the copy number of rDNA units in *H. vulgare*. Instead, the dataset is to illustrate the existence of scattered copies of ITS1 located outside the main rDNA arrays (Figure).

Table. The distribution of ITS1 on *H. vulgare* chromosomes. The ITS1 regions located outside the arrays of complete rDNA units are highlighed in yellow.

Chromosome	Pairwise identity (%)	Hit end	Hit start	Query coverage (%)	located within incomplete units outside arrays
chr1H	99.1	118033793	118033906	52.53	yes
chr1H	97.7	130717592	130717808	100.00	no
chr1H	97.2	130741159	130741375	100.00	no
chr1H	96.8	130755471	130755687	100.00	no
chr1H	98.2	130766271	130766486	100.00	no
chr1H	98.2	130779210	130779426	100.00	no
chr1H	97.7	130790869	130791085	100.00	no
chr1H	99.1	130791705	130791812	49.77	no
chr1H	98.2	130802686	130802902	100.00	no
chr1H	97.2	130816986	130817202	100.00	no
chr1H	98.6	130828393	130828609	100.00	no
chr1H	98.6	130840299	130840515	100.00	no
chr1H	96.0	130853737	130853786	23.04	no
chr1H	97.1	130853783	130853885	47.47	no
chr1H	96.8	130868719	130868935	100.00	no
chr1H	96.3	130882215	130882431	100.00	no
chr1H	98.2	130893786	130894002	100.00	no
chr1H	96.8	130918979	130919195	100.00	no
chr1H	97.2	130929854	130930070	100.00	no
chr1H	97.7	130943355	130943571	100.00	no
chr1H	97.7	130953888	130954104	100.00	no
chr1H	95.9	130965504	130965720	100.00	no
chr1H	98.2	130981236	130981452	100.00	no
chr1H	98.2	130993138	130993354	100.00	no
chr1H	98.7	131006625	131006781	72.35	no
chr1H	98.6	131006987	131007203	100.00	no
chr1H	96.0	131019097	131019146	23.04	no
chr1H	97.1	131019143	131019245	47.47	no
chr1H	96.8	131032943	131033159	100.00	no
chr1H	96.8	131047175	131047391	100.00	no
chr1H	97.0	131047600	131047665	30.41	no
chr1H	97.7	131060536	131060752	100.00	no
chr1H	97.2	131078244	131078458	99.08	no
chr1H	98.2	131092032	131092248	100.00	no
chr1H	97.2	131105356	131105572	100.00	no
chr1H	96.8	131116664	131116880	100.00	no
chr1H	98.6	131130317	131130533	100.00	no
chr1H	98.1	131143452	131143666	99.08	no
chr1H	98.6	131156571	131156787	100.00	no
chr1H	96.8	131168318	131168533	100.00	no
chr1H	98.6	131179320	131179536	100.00	no
chr1H	97.7	131192299	131192515	100.00	no
chr1H	98.1	131202474	131202689	99.54	no
chr1H	97.7	131213129	131213345	100.00	no
chr1H	95.4	131230249	131230033	100.00	no

chr1H	97.7	131241817	131242033	100.00	no
chr1H	98.1	131253918	131254133	99.54	no
chr1H	98.2	131266727	131266943	100.00	no
chr1H	97.2	131279051	131279267	100.00	no
chr1H	92.2	131322894	131323110	100.00	yes
chr1H	91.7	131399299	131399515	100.00	yes
chr1H	89.9	131427311	131427527	100.00	yes
chr1H	84.8	131464574	131464774	100.00	yes
chr1H	89.8	131512122	131512336	99.08	yes
chr1H	97.2	184756238	184756344	49.31	yes
chr2H	94.9	160175713	160175497	100.00	yes
chr2H	95.4	160214061	160213845	100.00	yes
chr2H	96.3	160214648	160214432	100.00	yes
chr2H	95.9	160238992	160238776	100.00	yes
chr2H	97.7	160253210	160252994	100.00	yes
chr2H	95.4	160265828	160265612	100.00	yes
chr2H	94.5	160290612	160290396	100.00	yes
chr4H	90.5	142551610	142551537	34.10	yes
chr4H	95.9	142569407	142569623	100.00	yes
chr4H	97.8	142581512	142581649	64.06	yes
chr4H	95.8	242301746	242301652	43.78	yes
chr5H	97.7	53056365	53056581	100.00	yes
chr5H	98.2	53079158	53079374	100.00	yes
chr5H	97.2	53091433	53091649	100.00	yes
chr5H	88.0	53105341	53105536	100.00	no
chr5H	98.2	53119146	53119362	100.00	no
chr5H	96.8	53130534	53130749	99.54	no
chr5H	94.5	53142845	53143058	100.00	no
chr5H	98.1	53153566	53153617	23.96	no
chr5H	97.2	53153897	53154113	100.00	no
chr5H	97.7	53161466	53161682	100.00	no
chr5H	96.8	53176106	53176322	100.00	no
chr5H	98.1	53189070	53189284	99.08	no
chr5H	98.2	53201529	53201745	100.00	no
chr5H	96.8	53210586	53210802	100.00	no
chr5H	96.8	53252110	53252326	100.00	no
chr5H	97.2	53267870	53268086	100.00	no
chr5H	96.8	53281184	53281400	100.00	no
chr5H	95.9	53293391	53293607	100.00	no
chr5H	95.4	53306561	53306776	99.54	no
chr5H	97.7	53320048	53320262	99.08	no
chr5H	97.7	53322759	53322975	100.00	no
chr5H	96.3	53335543	53335759	100.00	no
chr5H	96.8	53363687	53363903	100.00	no
chr5H	94.0	53414063	53414277	99.08	no
chr5H	94.9	53432443	53432658	100.00	yes
chr5H	88.9	123171296	123171080	100.00	yes
chr5H	91.1	476032091	476032134	20.74	yes
chr5H	89.5	476042135	476042220	39.63	yes
chr6H	100.0	82852352	82852568	100.00	yes
chr6H	99.5	82838656	82838872	100.00	yes
chr6H	98.5	89373151	89373217	30.88	yes

chr7H	89.5	13931162	13931030	60.83	yes
chr7H	96.3	192583343	192583558	100.00	yes
chr7H	96.3	192592889	192593105	100.00	yes
chr7H	97.2	192604438	192604654	100.00	yes
chr7H	100.0	192619066	192619110	20.74	yes
chr7H	96.3	192619619	192619835	100.00	yes
chr7H	96.8	192635110	192635326	100.00	yes
chrUn	96.3	30824651	30824866	100.00	yes
chrUn	93.5	30837604	30837819	100.00	yes
chrUn	100.0	36758578	36758653	35.02	yes
chrUn	100.0	36758767	36758857	41.94	yes
chrUn	99.1	38023786	38023570	100.00	yes
chrUn	99.5	38353245	38353461	100.00	yes
chrUn	100.0	38529032	38528816	100.00	yes
chrUn	99.1	39031433	39031217	100.00	yes
chrUn	100.0	39677024	39677236	98.16	yes
chrUn	100.0	41003505	41003603	45.62	yes
chrUn	100.0	43035689	43035555	62.21	yes
chrUn	99.1	45211201	45210985	100.00	yes
chrUn	100.0	45717289	45717073	100.00	yes
chrUn	100.0	45717507	45717338	78.34	yes
chrUn	98.6	49431497	49431281	100.00	yes
chrUn	97.7	53763542	53763326	100.00	yes
chrUn	100.0	56154155	56154217	29.03	yes
chrUn	100.0	56154326	56154482	72.35	yes
chrUn	100.0	57271033	57270940	43.32	yes
chrUn	99.1	60794108	60794324	100.00	yes
chrUn	99.1	60867655	60867439	100.00	yes
chrUn	97.2	62239316	62239532	100.00	yes
chrUn	98.7	64590143	64590218	35.02	yes
chrUn	99.1	64590227	64590443	100.00	yes
chrUn	100.0	65238528	65238610	38.25	yes
chrUn	100.0	67807621	67807664	20.28	yes
chrUn	97.8	67807751	67807933	84.33	yes
chrUn	100.0	67935327	67935373	21.66	yes
chrUn	100.0	67935463	67935630	77.42	yes
chrUn	99.5	67978602	67978818	100.00	yes
chrUn	99.1	68213295	68213511	100.00	yes
chrUn	100.0	68388221	68388409	87.10	yes
chrUn	99.4	69060395	69060554	73.73	yes
chrUn	99.5	71431207	71430991	100.00	yes
chrUn	100.0	71443700	71443802	47.47	yes
chrUn	99.1	72098503	72098287	100.00	yes
chrUn	100.0	72713941	72714007	30.88	yes
chrUn	100.0	72714049	72714236	86.64	yes
chrUn	99.5	72756180	72755964	100.00	yes
chrUn	100.0	73000513	73000729	100.00	yes
chrUn	100.0	73000792	73000915	57.14	yes
chrUn	99.5	73143261	73143045	100.00	yes
chrUn	100.0	73163240	73163038	93.55	yes
chrUn	99.5	74180564	74180348	100.00	yes
chrUn	99.2	74303371	74303498	58.99	yes

chrUn	99.1	74303504	74303719	99.54	yes
chrUn	100.0	74439128	74438912	100.00	yes
chrUn	94.8	74829201	74829316	53.46	yes
chrUn	95.8	74829319	74829485	76.96	yes
chrUn	100.0	75041631	75041447	85.25	yes
chrUn	99.5	75158524	75158308	100.00	yes
chrUn	100.0	75339679	75339741	29.03	yes
chrUn	99.5	75968228	75968444	100.00	yes
chrUn	100.0	76060762	76060578	85.25	yes
chrUn	99.1	76187899	76187683	100.00	yes
chrUn	100.0	76190400	76190309	42.40	yes
chrUn	99.5	76319376	76319592	100.00	yes
chrUn	100.0	76411199	76411037	75.12	yes
chrUn	100.0	76458032	76458137	48.85	yes
chrUn	100.0	76458158	76458366	96.31	yes
chrUn	100.0	76654002	76653890	52.07	yes
chrUn	100.0	76663802	76663676	58.53	yes
chrUn	100.0	76989106	76988961	67.28	yes
chrUn	99.5	77048496	77048712	100.00	yes
chrUn	100.0	77411617	77411512	48.85	yes
chrUn	99.5	77565240	77565024	100.00	yes
chrUn	100.0	78229509	78229425	39.17	yes
chrUn	100.0	79364600	79364492	50.23	yes
chrUn	99.5	80396979	80396765	99.08	yes
chrUn	99.5	80688095	80688311	100.00	yes
chrUn	99.1	80963145	80963030	53.46	yes
chrUn	100.0	81894023	81894109	40.09	yes
chrUn	99.5	83153075	83153291	100.00	yes
chrUn	100.0	84025368	84025152	100.00	yes

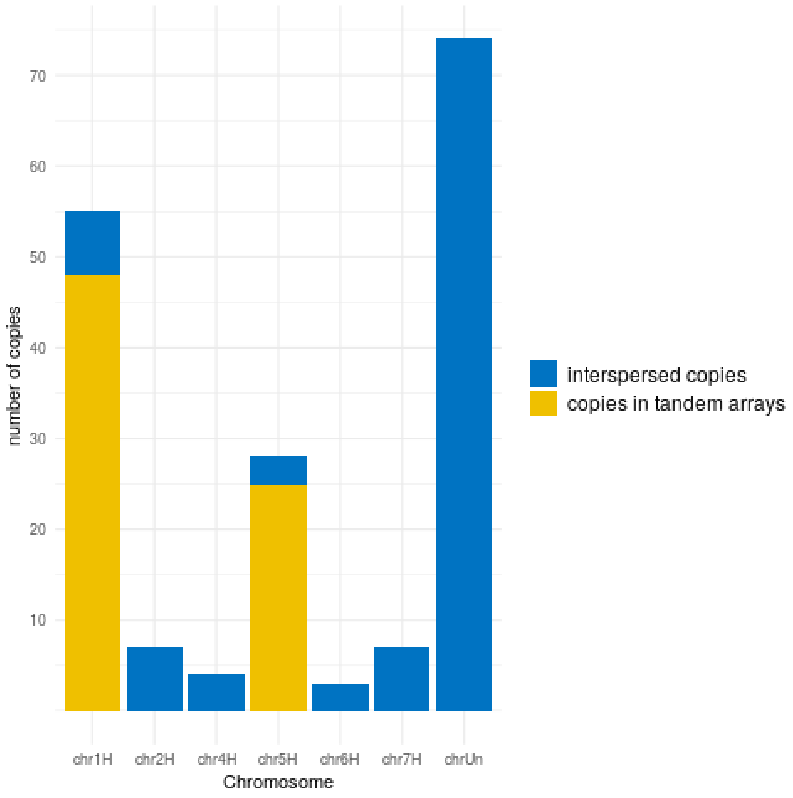

Figure. The distribution of ITS1 on *H. vulgare* chromosomes.

References

Monat, C., Padmarasu, S., Lux, T., Wicker, T., Gundlach, H., Himmelbach, A. et al. (2019). TRITEX: Chromosome-scale sequence assembly of Triticeae genomes with open-source tools. *Genome Biology*. 20: 284. doi:10.1186/s13059-019-1899-5.

Nelson et al. (2019). Mechanisms of rDNA copy number maintainance. *Trends in Genetics*. 35: 734–742.
